# Guidelines for Perioperative Care in Elective Abdominal and Pelvic Surgery at Primary and Secondary Hospitals in Low–Middle-Income Countries (LMIC’s): Enhanced Recovery After Surgery (ERAS) Society Recommendation

**DOI:** 10.1007/s00268-022-06587-w

**Published:** 2022-05-31

**Authors:** Ravi Oodit, Bruce M. Biccard, Eugenio Panieri, Adrian O. Alvarez, Marianna R. S. Sioson, Salome Maswime, Viju Thomas, Hyla-Louise Kluyts, Carol J. Peden, Hans D. de Boer, Mary Brindle, Nader K. Francis, Gregg Nelson, Ulf O. Gustafsson, Olle Ljungqvist

**Affiliations:** 1 Division of Global Surgery, University of Cape Town, Anzio Road, Observatory, Cape Town, Western Cape South Africa; 2 Department of Anesthesia and Perioperative Medicine, Groote Schuur Hospital, University of Cape Town, Anzio Road, Observatory, Cape Town, Western Cape South Africa; 3 Division of General Surgery, Groote Schuur Hospital, University of Cape Town, Anzio Road, Observatory, Cape Town, Western Cape South Africa; 4 Anesthesia Department, Hospital Italiano de Buenos Aires, Teniente General Juan Domingo Peron, 4190, C1199ABB Beunos Aires, Argentina; 5 Head Section of Medical Nutrition, Department of Medicine and ERAS Team, The Medical City, Ortigas Avenue, Manila, Metro Manila Philippines; 6 Department of Obstetrics and Gynaecology, Tygerberg Hospital and University of Stellenbosch, Francie Van Zyl Drive, Parow, Cape Town, Western Cape South Africa; 7 Department of Anaesthesiology, Sefako Makgatho Health Sciences University, Medunsa, Molotlegi Street, P.O. Box 60, Ga-Rankuwa, Pretoria, 0204 Gauteng South Africa; 8 Keck School of Medicine, University of Southern California, 1975 Zonal Ave, Los Angeles, CA 90033 USA; 9 Perelman School of Medicine, University of Pennsylvania, Philadelphia, PA 19104 USA; 10 Department of Anesthesiology, Pain Medicine and Procedural Sedation and Analgesia, Martini General Hospital Groningen, Van Swietenplein 1, 9728 NT Groningen, The Netherlands; 11 Cumming School of Medicine, University of Calgary, London, Canada; 12 Alberta Children’s Hospital, Calgary, Canada; 13 Safe Systems, Ariadne Labs, Stockholm, USA; 14 EQuIS Research Platform, Orebro, Canada; 15 Division of Surgery and Interventional Science- UCL, Gower Street, London, WC1E 6BT UK; 16 Department of Obstetrics & Gynecology, University of Calgary, 1331 29 St NW, Calgary, AB T2N 4N2 Canada; 17 Department of Clinical Sciences, Division of Surgery, Danderyd Hospital, Karolinska Institutet, Entrevägen 2, 19257 Stockholm, Danderyd Sweden; 18 School of Medical Sciences, Department of Surgery, Örebro University, 701 85 Örebro, Sweden

## Abstract

**Background:**

This is the first Enhanced Recovery After Surgery (ERAS®) Society guideline for primary and secondary hospitals in low–middle-income countries (LMIC’s) for elective abdominal and gynecologic care.

**Methods:**

The ERAS LMIC Guidelines group was established by the ERAS® Society in collaboration with different representatives of perioperative care from LMIC’s. The group consisted of seven members from the ERAS® Society and eight members from LMIC’s. An updated systematic literature search and evaluation of evidence from previous ERAS® guidelines was performed by the leading authors of the Colorectal (2018) and Gynecologic (2019) surgery guidelines (Gustafsson et al in World J Surg 43:6592–695, Nelson et al in Int J Gynecol Cancer 29(4):651–668). Meta-analyses randomized controlled trials (RCTs), prospective and retrospective cohort studies from both HIC’s and LMIC’s were considered for each perioperative item. The members in the LMIC group then applied the current evidence and adapted the recommendations for each intervention as well as identifying possible new items relevant to LMIC’s. The Grading of Recommendations, Assessment, Development and Evaluation system (GRADE) methodology was used to determine the quality of the published evidence. The strength of the recommendations was based on importance of the problem, quality of evidence, balance between desirable and undesirable effects, acceptability to key stakeholders, cost of implementation and specifically the feasibility of implementing in LMIC’s and determined through discussions and consensus.

**Results:**

In addition to previously described ERAS® Society interventions, the following items were included, revised or discussed: the Surgical Safety Checklist (SSC), preoperative routine human immunodeficiency virus (HIV) testing in countries with a high prevalence of HIV/AIDS (CD4 and viral load for those patients that are HIV positive), delirium screening and prevention, COVID 19 screening, VTE prophylaxis, immuno-nutrition, prehabilitation, minimally invasive surgery (MIS) and a standardized postoperative monitoring guideline.

**Conclusions:**

These guidelines are seen as a starting point to address the urgent need to improve perioperative care and to effect data-driven, evidence-based care in LMIC’s.

## Introduction

The Lancet Commission and the Global Surgery Foundation in 2015 highlighted the urgent need to improve the access gap to safe surgery and anesthesia for essential surgical services in low- and middle-income countries (LMIC’s) [1–3]. However, in addition to the access gap, the International Surgical Outcomes Study (ISOS) and the African Surgical Outcomes Study (ASOS) highlighted the urgent need to address the quality gap in perioperative care in LMIC’s [4, 5]. To address the quality gap and to ensure sustainable change, perioperative care needs to be evidence-based, standardized, patient centered, cost effective, efficient, and supported by good outcome data [6].

The effective implementation of an Enhanced Recovery After Surgery (ERAS®) program, when combined with a compliance of approximately 70% or greater, has demonstrated significant improvement in the quality of perioperative care and patient outcomes in high-income countries (HIC’s), but to date the program has had only limited application in LMIC`s [7]. The ERAS perioperative guidelines should be practicable in tertiary hospitals in LMIC’s, staffed by subspecialists working with a perioperative multidisciplinary team. They may, however, be less feasible in smaller primary and secondary care level hospitals [8]. These smaller facilities are where a significant volume of surgery is carried out in LMICs and are commonly staffed by doctors working across multiple disciplines without specialist training, with little or no support from a perioperative multidisciplinary team(MDT), no access to a perioperative nurse coordinator and a lack of quality patient outcomes data.

This paper reports on the exploratory phase of a project that aims to identify key ERAS interventions in elective abdominal and pelvic surgery which can be used as original or modified interventions in a low resource clinical environment. This project aims to provide a simplified LMIC ERAS platform with a balanced and standardized perioperative practice framework that confers clinical benefit to patients and is relatively easy to manage. The adapted guidelines presented here are meant to be tested and evaluated within this context. It is expected that such testing will drive a process of re-evaluation and further updates.

## Methods

The ERAS LMIC Guidelines group was established by the ERAS® Society in collaboration with different representatives of perioperative care from LMIC’s. The group consisted of seven members from the ERAS® Society and eight members from LMIC’s. The main purpose of the group was to review the current evidence and recommendations in colorectal and gynecologic surgery from the ERAS® Society for relevance in LMIC’s and to identify any new items that may be contextually relevant in LMIC’s. An updated systematic literature search and evaluation of evidence from previous ERAS® guidelines was performed by the leading authors of the Colorectal (2018) and Gynecologic (2019) surgery guidelines [9, 10]. PubMed, Embase and Cochrane databases were used. Key words included: pre-, intra- and postoperative ERAS care elements (Table 1). The literature search included full text English language articles. Meta-analyses randomized controlled trials (RCTs), prospective and retrospective cohort studies from both HIC’s and LMIC’s were considered for each perioperative item. The World Bank classification of HIC’s and LMIC’s was used [11].The members in the LMIC group then applied the current evidence and adapted the recommendations for each intervention as well as identifying possible new items relevant to LMIC’s.

**Table 1:** Essential perioperative care interventions for elective abdominal and gynecologic surgery at primary and secondary hospitals in LMIC’s

	Definition	Grade of Evidence	Grade of Recommendation
*Preoperative care items*			
Education	The patient and a relative or care giver should receive preoperative education in oral, written and or pictorial format	Moderate	Strong
Optimization	In addition to clinical cardiorespiratory assessment, patients should be screened for smoking, alcohol usage, hypertension, diabetes and anemia and have a nutritional assessment, preoperative HIV testing in countries with high HIV/AIDS prevalence and delirium screening	High	Strong
Selective use of mechanical bowel preparation(MBH)	No routine bowel preparation for patients undergoing elective colonic or gynecologic surgery	High	Strong
Fasting	Oral intake of clear fluids up to 2 h and a light meal up to six hours before induction. After a full meal (including meat, fatty and fried foods) 8 or more hours may be required	High	Strong
Carbohydrate (CHO) drink	A complex carbohydrate drink, 400 ml with 50 g CHO (12 g/100 ml), osmolality of < 300 mOsm/kg, should be given 2 h before surgery for elective patients	Moderate	Strong
Premedication	Avoid routine use of premedication. Consider a short-acting anxiolytic in patients with severe anxiety	Low	Strong
*Intraoperative care items*			
Surgical safety checklist	Routine use of the 19 checklist items and its three pause points	High	Strong
Antimicrobial prophylaxis	A first-generation cephalosporin is recommended. Antibiotics should be administered within 1 h of incision. Antibiotic prophylaxis is not recommended in the postoperative period	High	Strong
Postoperative nausea and vomiting (PONV) prophylaxis	All patients should have a risk assessment for PONV. High-risk patients should receive 2–3 antiemetics. Continue postoperatively as required	High	Strong
Venous thromboembolism (VTE) prophylaxis	A combination of a compression stocking and/or intermittent pneumatic compression together with either a LMWH or unfractionated heparin should be used and continued in hospital	High	Strong
Standard anesthesia protocol	Short-acting anesthetic agents, lung-protective ventilation, and complete reversal of neuromuscular blockade,	High	Strong
Normothermia	Core temperature should be maintained at > 36 °C. Active warming should be carried out in all patients in operations lasting longer than 30 min	High	Strong
Multimodal opioid sparing analgesia	Short-acting opioid sparing analgesia combined with local and regional blocks. In open abdominal surgery a mid-thoracic epidural analgesia should be used. Spinal analgesia and local blocks can be used in minimally invasive surgery	High	Strong
Fluid balance	Near- zero fluid balance. Intravenous treatment should be discontinued day1. Patients should be encouraged to drink when fully recovered and offered an oral diet within 4 h after surgery	High	Strong
Minimally invasive surgery (MIS)	MIS is preferred for appropriate patients where the resources and expertise are available	High	Strong
Avoid nasogastric tubes (NGT) and drains	The routine use of nasogastric tubes and drains is not recommended	High	Strong
*Postoperative care items*			
Early oral feeding	Oral fluids as soon as the patient is lucid after surgery and solids after 4 h	Moderate	Strong
Early mobilization	30 min on the day of surgery and 6 h/day thereafter	Moderate	Strong
Multimodal opioid sparing analgesia	A combination of paracetamol and NSAID given orally with additional use of non-opioid drugs if needed. Opioid containing drugs should be used as a last resort and in low doses	High	Strong
Urinary catheter	Foleys catheter should be removed in the majority of cases within 24 h after surgery and individualized in patients with high risk of retention	High	Strong
Audit and evaluation	Continuous audit of processes of care, compliance to guidelines, and outcomes is recommended	High	Strong
Tailored monitoring, evaluation and escalation of care	Key parameters to monitor include respiratory and heart rate, blood pressure, oxygen saturation, level of consciousness and surgical site. A tailored, postoperative monitoring, evaluation and escalation of care pathway is recommended	Moderate	Strong

### Quality assessment

The Grading of Recommendations, Assessment, Development and Evaluation system (GRADE) methodology was used to determine the quality of the published evidence with the highest available evidence from randomized controlled trials, meta-analyses and large cohort series [12]. The quality of the evidence for each publication was graded to be low, moderate or high through an assessment of risk of bias within individual studies, publication bias across studies, precision and consistency. The strength of the recommendations was based on importance of the problem, quality of evidence, balance between desirable and undesirable effects, acceptability to key stakeholders, cost of implementation and specifically the feasibility of implementing in LMIC’s and determined through discussions and consensus.

### Evidence-to-decision framework

Each original and modified ERAS intervention and its supporting evidence was reviewed by the entire working group to determine the quality of recommendation. In addition, the LMIC group was asked to discuss and evaluate additional items not included in previous ERAS guidelines based on new evidence or unique needs in LMIC’s. These items were then discussed by the entire working group and agreement reached for possible inclusion. The LMIC working group provided recommendations for each item as strong or weak, a conditional recommendation or no recommendation. This was then presented, discussed and agreement reached by the entire working group.

## Results

The definitions, grade of evidence and recommendations of perioperative care items are summarized in Table1. In addition to previously described ERAS® Society interventions, the following items were included, revised or discussed: the Surgical Safety Checklist (SSC), preoperative routine human immunodeficiency virus (HIV) testing in countries with a high prevalence of HIV/AIDS (CD4 and viral load for those patients that are HIV positive), delirium screening and prevention, COVID 19 screening, VTE prophylaxis, immuno-nutrition, prehabilitation, minimally invasive surgery (MIS) and a standardized postoperative monitoring guideline.

### Preoperative education

The aim of preoperative education is to establish clear expectations about the surgical and anesthetic care plan, ensure that the patient and their families understand their role in successful recovery, and to establish discharge plans preoperatively. Preoperative education has been shown to reduce patient anxiety, pain, nausea and vomiting and increase patient satisfaction [13, 14]. Patients managed under an ERAS pathway are discharged in an intermediate phase of recovery, with the recovery process expected to extend into the home setting. Managing this transition and ensuring that it is tailored to the individual patient needs is crucial to ensure that patients and care givers feel safe and reassured and have clear and concise emergency contact details and transport plans. This is particularly important in LMIC’s where a frequent challenge is long travel distances and poor access to transport.

Ideally the patient and a relative or care giver should receive preoperative education in oral, written and or pictorial format. Preoperative education is usually carried out by the ERAS nurse coordinator soon after the decision is made to operate. This allows the patient, their families and care givers sufficient time to process the information. A nurse coordinator and wider multidisciplinary team (MDT)are unlikely to be available in the primary and secondary hospital setting in LMIC’s and this role will need to be fulfilled by an elected health care professional.

Summary: Preoperative education is given to the patient and a relative or care giver in oral, written and or pictorial format.

**Table:** 

Evidence level	moderate
Recommendation grade	strong

### Preoperative optimization

In addition to clinical cardiorespiratory assessment, patients should be screened for smoking, alcohol usage, undiagnosed hypertension, diabetes, anemia, nutritional assessment, routine preoperative HIV testing in countries with high prevalence of HIV and cognitive assessment. The long lead time from scheduling to proceeding with surgery in LMIC’s could provide an opportunity to optimize patients preoperatively.

#### Smoking cessation

Patients who smoke have an increased risk of intra- and postoperative complications [15]. Smoking cessation of 4 to 8 weeks is necessary to reduce respiratory and wound-healing complications [16]. It is unclear whether short-term, less than 4 weeks smoking cessation, reduces the risk of postoperative respiratory complications.

Summary: Smoking cessation is recommended, preferably 4 weeks or more before the operation.

**Table:** 

Evidence	high
Recommendation	strong

#### Avoiding alcohol abuse

Alcohol has a negative effect on the catabolic stress response and drives immune suppression in the perioperative period. In a recent systematic review and meta-analysis, the consumption of more than two units of alcohol per day was associated with an increase in postoperative infections [17]. In a subsequent sub-analysis and in a 2012 Cochrane review, it was also shown that cessation of alcohol usage for a minimum of 4 weeks was associated with fewer complications but had no impact on mortality or length of stay[18].

Summary: Preoperative abstinence of alcohol for 4 weeks prior to surgery is recommended.

**Table:** 

Evidence	moderate
Recommendation	strong

#### Anemia

Preoperative anemia is associated with an increased risk of postoperative complications, increased rate of blood transfusion and mortality and may worsen long-term oncology outcomes.[19–21]. Anemia is common in patients presenting for surgery with reported prevalence rates of up to 31.1% [19]. Patients scheduled for surgery may have many factors causing anemia. These include acute or chronic blood loss, iron, vitamin B12 or folate deficiency and anemia of chronic disease or a combination of these [22].

Patients with anemia should be investigated preoperatively. The cause and type identified, and the anemia corrected preoperatively [22]. In patients with iron deficiency anemia, oral or intravenous iron can be used [22, 23]. Oral iron is inexpensive and easy to administer but may be poorly tolerated, especially in patients with gastrointestinal cancer. Intravenous iron has a low risk of adverse reactions and is more effective than oral iron at restoring hemoglobin concentrations in both iron deficiency anemia and anemia of chronic disease [22, 24]. Blood transfusion should be avoided as it has a potential for significant short- and long-term complications and is a scare resource in most LMIC’s [20].

The recent PREVENTT trial showed that the use of intravenous iron in patients with all types of anemia before major open elective abdominal surgery increased hemoglobin concentrations before surgery but did not reduce the frequency of blood transfusion, mortality, in hospital complications, length of stay or quality of life relative to a placebo [22]. However, there was a reduced risk of readmission to hospital for complications in those patients who received intravenous iron.

A 2021 systematic review, which included 10 RCTs and 1039 participants, showed that preoperative IV iron supplementation decreased blood transfusion by 16% and was not associated with increased incidence of any adverse effects across the groups [24].

Summary:

Routine preoperative screening and treatment of anemia is recommended

**Table:** 

Evidence	moderate
Recommendation	strong

Restrictive blood transfusion practice is recommended

**Table:** 

Evidence	high
Recommendation	strong

#### Routine preoperative nutritional screening

Patients with unintentional weight loss of 5% of body mass over 3 months or 10% over 6 months suffer from an increased risk of postoperative complications, mortality, and worse long-term oncology outcomes [25]. In malnourished patients, nutritional supplementation is associated with a reduction of infectious complications and anastomotic leaks [25].

There is no gold standard on how to assess nutritional status accurately preoperatively [26]. Available tools include: the Nutritional Risk Screening score (NRS 2002), the Subjective Global Assessment (SGA), the Patient Generated Subjective Global Assessment (PG- SGA) and the Malnutrition Universal Screening Tool (MUST) [26–28]. Patients at increased risk should receive nutritional treatment preferably using the oral route for a period of at least 7–10 days prior to surgery [25].

Summary:

Routine preoperative nutritional screening and assessment as needed is recommended

**Table:** 

Evidence	low
Recommendation	strong

Preoperative nutritional assessment and support in the malnourished patient is recommended.

**Table:** 

Evidence	moderate
Recommendation	Strong

#### Routine HIV screening

HIV continues to be a major global public health issue [29]. There were an estimated 37.7 million people living with HIV at the end of 2021, the majority living in LMIC’s [30]. It is estimated that between 20 and 25% of HIV positive people will require surgery in their lifetime [31]. Although there is good quality evidence that patients with HIV/AIDS have significant and consistently poor postoperative outcomes after any major surgery, the data are unclear in asymptomatic HIV positive patients due to the low quality of publications [30–32]. HIV infection can negatively impact on postoperative patient outcomes through multiple pathways and include: CD4 and macrophage dysfunction, compromised gut associated lymphoid tissue (GALT), including those patients on optimal treatment, and a higher risk of comorbidities (chronic respiratory, liver, and renal disease, anemia, hepatitis. In addition, antiretroviral treatment (ART) can cause renal and hepatotoxicity [30, 31, 33]. Information on immunological vulnerability could guide the timing and type of procedure and potentially reduce postoperative complications.

Routine screening for HIV including CD4 counts and viral loads on those living with HIV is not a prerequisite before surgery in most LMIC’s. However, routine preoperative testing has the potential to identify patients with undiagnosed HIV infection and among those with known HIV identify those on suboptimal ART or poorly adherent to treatment. These patients can be optimized preoperatively and possibly improve their postoperative surgical outcomes. For patients on antiretroviral treatment (ART), preventing postoperative ileus and commencing early oral feeding, as recommended in these guidelines will facilitate early recommencement of ART after surgery.

Given the potential multi-layer negative impact on postoperative outcomes in HIV positive patients, the routine testing and optimizing of patients preoperatively in countries with a high prevalence is recommended but needs to be subjected to robust investigation.

Summary:

Routine HIV screening in countries with high prevalence is recommended.

**Table:** 

Evidence	moderate
Recommendation	strong

Optimizing ART before surgery.

**Table:** 

Evidence	low
Recommendation	strong

#### Delirium screening and prevention

Delirium is an acute neuro-behavioral syndrome caused by abnormal neuronal activity and neuroinflammation in response to systemic disturbances, such as surgery, stress and infection. Delirium can present with agitation, or as quiet delirium, where the patient is still and withdrawn, and this latter type is more common and frequently missed. Early detection and management are vital, as studies show that the longer a patient remains delirious the greater the harm which includes higher mortality, morbidity, complications and length of stay [34]. There is little data on the perioperative incidence of delirium in LMIC’s and further research is required [35]. Surgical patients at increased risk are those over 65 years of age and/or those with a preoperative cognitive dysfunction.

Systematic reviews, expert consensus opinion, guidelines on best practice and low-cost interventions and preventative measures are available [36–39]. The 4AT test and the Hospital Elder Life Program (HELP) have been shown in randomized controlled trials and systematic reviews to significantly reduce the incidence of delirium with simple actions such as increasing the presence of family and friends, regular orientation, and early mobilization [38–41]. A recent international expert consensus review from the American Society of Anesthesiology (ASA) includes the following: education and training for the multidisciplinary care team on screening, detection and prevention of delirium, non-pharmacologic interventions such as return of hearing aids, and promoting the presence of family, optimal multimodal pain control and selective use of antipsychotics and anxiolytics; all these actions should be feasible in resource-constrained environments. [37]. Regular delirium screening should be carried out prior to discharge from the recovery room, and then daily until day five or discharge. [37].

Summary: Delirium screening should be performed, and simple strategies to reduce incidence in at-risk patients are recommended.

**Table:** 

Evidence	high
Recommendation	strong

#### COVID 19

Guidelines specific to the current COVID 19 pandemic was discussed for possible inclusion. However, it was clear this has been effectively covered in other publications, that the evidence continues to evolve and that perioperative care teams do need to know how to deliver surgical services safely and effectively during any pandemic [42]. It has thus not been included as a specific care item in these guidelines. However, it is essential that perioperative care teams actively participate in formulating national pandemic plans. The key areas to consider in these plans include training and support for the perioperative MDT, a shared decision-making approach to reduce non-urgent procedures and prioritizing emergency services and plans to address the inevitable backlog.

### Selective use of mechanical bowel preparation

Mechanical bowel preparation has traditionally been used with the belief that it would reduce surgical site infection and anastomotic leaks. However, its use is associated with pre-operative dehydration, electrolyte abnormalities, patient dissatisfaction, an interrupted sleep the night before surgery and increased patient anxiety [43].

The current evidence does not support the routine use of mechanical bowel preparation for patients undergoing elective colonic or gynecologic surgery. There may be some benefit in patients undergoing elective low rectal surgery or having a planned elective de-functioning ileostomy or colostomy [44]. However, these elective procedures are highly unlikely to be carried out at a primary and secondary hospital in a LMIC’s.

Summary: Selective use of mechanical bowel preparation is recommended.

**Table:** 

Evidence	high
Recommendation	strong

### Preoperative fasting

Patients are traditionally kept nil per mouth (NPO) from midnight before their planned surgery, despite a lack of quality evidence to support this practice, to potentially reduce the risk of aspiration of gastric contents at induction of anesthesia.

However, preoperative fasting results in a prolonged period without fluids or nutrition. Multiple meta-analyses have demonstrated that healthy adults undergoing elective surgery, oral intake of clear fluids up to 2 h and a light meal up to six hours before induction does not increase the risk of aspiration [45]. After a full meal (including meat, fatty and fried foods) 8 or more hours may be required.

Summary: Free intake of clear fluids up to 2 h and a light meal until 6 h before induction of anesthesia is recommended unless specific contraindications exist.

**Table:** 

Evidence	high
Recommendation	strong

### Preoperative carbohydrate (CHO) drink

Major abdominal surgery, especially open surgery, is associated with insulin resistance. This drives the catabolic response and results in an increased risk of complications [46]. Several systematic reviews and meta-analyses have demonstrated that tailored, preoperative commercially available CHO drinks given two hours before surgery reduce postoperative insulin resistance, particularly in open abdominal surgery [47]. A complex carbohydrate (polysaccharide, maltodextrin) drink, 400 ml with 50 g CHO (12 g/100 ml), osmolality of < 300 mOsm/kg, is reliably emptied from the stomach within 2 h and elicits the intended insulin release to change metabolism from a fasted to a fed state [46]. Although “sports” drinks are freely available and relatively inexpensive, they contain only half the concentration of CHO and are not intended to release insulin and impact on perioperative metabolism. The data available for diabetic patients are not sufficient to give any clear recommendation.

Summary: Intake of CHO drinks designed for preoperative use is recommended to non-diabetic patients.

**Table:** 

Evidence	moderate
Recommendation	strong

### Preoperative sedation

The concern with the routine use of either long-acting sedatives is the delay in return to full psychomotor function, early mobilization and feeding postoperatively and an increased risk of delirium [48].

Summary: Avoidance of the routine use of preoperative anxiolytic drugs is recommended.

**Table:** 

Evidence level	low
Recommendation	strong

### Surgical safety checklist (SSC)

The World Health Organization published the SSC in 2008 with the aim to improve the safety of patients undergoing surgery [49]. This checklist of 19 items has three pause points used by the surgical team to confirm that appropriate actions are taken in the perioperative period to maintain patient safety and ensure there is shared understanding between the team members. The use of the SSC has been shown to significantly reduce perioperative morbidity and mortality in LMIC’s as well as in HIC settings [50]. It is one of the most affordable and sustainable tools for reducing deaths from surgery in LMIC’s [50]. Despite evidence of effectiveness, the acceptance and adoption of the SSC remain poor (20–40%) in LMIC’s [51]. The reasons are multifactorial and include a lack of resources and infrastructure for implementation, monitoring and evaluation. Adding the SSC to the ERAS implementation program could provide the platform for improved compliance to the SSC, improve patient safety and possibly assist in addressing the urgent need to reduce perioperative morbidity and mortality in LMIC’s [7].

Summary: Routine use of the SSC is recommended.

**Table:** 

Evidence	high
Recommendation	strong

### Antimicrobial prophylaxis

Surgical site infections (SSI) are defined as infections of the surgical incision or organ space that develop within 30 days of surgery. SSI’s are common occurring, with rates up to 23.5% reported in patients undergoing a laparotomy [52]. SSI occurs more commonly in LMIC than in HIC’s and substantially increases the overall risk of financial catastrophe for patients in LMIC’s [53]. Many studies and meta-analyses, including a 2014 Cochrane review, have demonstrated the benefit of intravenous antibiotic prophylaxis in reducing SSI [54].

A first-generation cephalosporin is recommended [54]. Cephalosporins have a broad spectrum of coverage, are inexpensive and have a low allergenic potential. Additional anaerobic coverage is recommended if the bowel is entered during pelvic surgery for cancer. Antibiotics should be administered within 1 h of incision to obtain the highest drug serum levels at incision. The dose should be increased in obese patients (BMI > 35). Additional dosing is not recommended in the postoperative period [55]. In patients with penicillin or cephalosporin allergy, a combination of intravenous clindamycin and gentamycin or a quinolone (e.g., ciprofloxacin) is recommended [55].

Summary: Prophylactic antibiotics should be administrated routinely within one hour of skin incision.

**Table:** 

Evidence	high
Recommendation	strong

### Nausea and vomiting prophylaxis

Postoperative nausea and vomiting (PONV) is common and occurs in 30% to 50% of all surgical patients and in up to 80% of high-risk patients [56]. Apart from patient dissatisfaction, PONV may result in delayed oral feeding, prolonged intravenous fluids, placement of a nasogastric tube, prolonged hospital stay and increase healthcare costs [57, 58].

All patients should have a risk assessment for PONV. There are several scoring systems, however the most used is the Apfel score [58]. Patients with a score of 1 should receive a combination of two drugs using first line antiemetics as prophylaxis. Patients with score of 2 or more should receive 2–3 antiemetics. If PONV persists despite adequate prophylaxis, a different class of drug should be used. Additional measures to reduce PONV include total intravenous anesthesia (TIVA) rather than volatile gases and multimodal analgesia rather than the liberal use of opioids. Recent studies have also shown benefit with the use of prophylactic analgesia with paracetamol [56, 59].

Summary: Routine screening for the risk of PONV and treatment given as needed is recommended.

**Table:** 

Evidence	high
Recommendation	Strong

#### Venous thromboembolism (VTE) prophylaxis

VTE is a potentially fatal postoperative complication. Additional VTE complications include pulmonary hypertension, cardiac failure and post-thrombotic syndrome. The following risk factors are known to be associated with a high risk for VTE: malignancy, obesity, pelvic surgery, pre-operative immunosuppressants, immobility and a hypercoagulable state.

Current evidence supports that every patient undergoing a major, elective abdominal or pelvic surgery should have VTE prophylaxis. For these cases, a combination of a compression stocking and/or intermittent pneumatic compression together with either a low molecular weight heparin (LMWH) or unfractionated heparin should be used and continued while in hospital. [60, 61].

There is ongoing debate on the use of extended (28 day) prophylaxis [62]. The incidence of post-discharge VTE (0.60–0.73%), DVT (0.29–0.48%) and PE (0.26–0.40%) is very low. [62]. For the elective procedures that are carried out in primary and secondary hospitals in LMIC’s that are in low-risk patients, the benefit of extended prophylaxis needs to be balanced against logistical challenges (e.g., travel, availability, refrigeration and cost.

Summary: Routine VTE prophylaxis while in hospital is recommended.

Stockings and pneumatic compression.

**Table:** 

Evidence	high
Recommendation	strong

Low molecular weight heparin.

**Table:** 

Evidence	high
Recommendation	strong

#### Standard anesthetic protocol

The key goals are to provide adequate amnesia, hypnosis, muscle relaxation, analgesia, maintain adequate circulation and oxygen delivery with little or no residual side effects with the aim to facilitate early recovery, mobilization and oral intake.

Specific objectives are to use short-acting anesthetic agents, lung-protective ventilation, achieve complete reversal of neuromuscular blockade.

Propofol has become the standard medication for induction of general anesthesia because of its rapid onset and recovery and reduced nausea and vomiting. There is no strong data to support the use of either volatile anesthetic gases (e.g., sevoflurane) or total intravenous anesthesia (TIVA) to maintain anesthesia [63]. Nitrous oxide is generally avoided due to its high risk of PONV and delayed return of bowel function [64]. Several intravenous anesthetic agents (e.g., dexmedetomidine, ketamine) may be used in combination with propofol to provide effective total intravenous anesthesia. Dexmedetomidine also reduces opioid requirements and ketamine potentially reduces chronic postoperative pain.

Whenever a neuromuscular blocker is administered, neuromuscular function must be monitored by observing the evoked muscular response to peripheral nerve stimulation. Ideally, this should be done at the hand muscles (not the facial muscles) with a quantitative (objective) monitor. Objective monitoring (documentation of train-of-four ratio ≥ 0.90) is the only method of assuring that satisfactory recovery of neuromuscular function has taken place [65].

The use of low tidal volumes (6-8mls/kg) with a positive end expiratory pressure of 6-8 cm of H_2_O has been shown in RCT’s to reduce pulmonary complications [66].

Summary: A standard anesthetic protocol that has the goal of using short-acting anesthetics, cerebral monitoring to improve recovery and reduce the risk for postoperative delirium, monitoring of the level and complete reversal of neuromuscular block is recommended.

**Table:** 

Evidence	low
Recommendation	strong

Complete reversal of neuromuscular block must be achieved before extubation and should be monitored using a peripheral nerve stimulator with a train of four ≥ 90%

**Table:** 

Evidence	high
Recommendation	strong

#### Normothermia

Intraoperative hypothermia (< 36 °C) is associated with increased blood loss, myocardial ischemia, cardiac arrhythmia as shivering increases oxygen consumption, increased risk of surgical site infections, and prolonged postoperative acute care units (PACU) and hospital stay [67].

Patients’ core temperature drops between 0.5 and 1.5 degrees within first 30 min after induction of general or neuro-axial anesthesia [68]. Active warming should be carried out in all patients in operations lasting longer than 30 min. Various methods to maintain normothermia are used including forced air blanket devices, underbody warming mattresses, prewarming and warmed intravenous fluid and anesthetic gases. The operating room temperature should be a minimum of 21 °C while the patient is exposed prior to commencing active warming [69].

Accurate measurement of temperature is essential. Core temperature measurement is ideal. Methods include nasopharyngeal temperature probe and zero heat flux thermometry.

Summary: Maintaining normothermia during and after surgery is recommended.

**Table:** 

Evidence	high
Recommendation	strong

#### Multimodal opioid sparing analgesia

An opioid sparing multimodal analgesia approach facilitates early mobilization and return of bowel function [7]. Short-acting opioid analgesics (e.g., remifentanil) during surgery do allow for consistent rapid recovery, but there is concern it may induce hyperalgesia [70]. Other agents to consider for analgesia during the procedure are ketamine, magnesium, steroids dexmedetomidine, lidocaine infusions and gabapentinoids [71–73]. There is limited data comparing the analgesic effect and side effects with other agents.

A mid-thoracic epidural (TEA), T7-10, is recommended for open abdominal surgery. It should be commenced before surgery and continued intraoperatively and ideally for 48–72 h postoperatively [74]. TEA has been shown to be better than systemic opioids in several RCT’s for open abdominal surgery [75, 76]. Additional benefits include reduction in the surgical catabolic response, insulin resistance, protein loss and time to return of bowel function [77]. The drawbacks with TEA include hypotension with a risk of fluid overload and urinary retention, and failure rates of up to 33.6% [77, 78]. If a patient with a TEA is hypotensive, it is important to confirm euvolemia and add a vasopressor before administering further intravenous fluid. A patient that is fluid responsive may not necessarily be fluid depleted [79].The signs of hypovolemia include tachycardia, hypotension, sweating, confusion and decreased capillary filling. Adequate postoperative expertise in epidural management needs to be available to assist with any pain management issues that may arise.

Current evidence does not support the use of epidural analgesia for MIS surgery [80, 81]. Alternative analgesic techniques in MIS surgery could include spinal analgesia, abdominal wall blocks, wound infusion catheters, lidocaine infusion, intraperitoneal local anesthetic and wound infiltration which all have been shown to be useful adjuncts to systemic analgesia [82–84].

Spinal analgesia has been shown to be effective in MIS colorectal surgery in an ERAS care pathway [81]. A combination of a long-acting opioid and a local anesthetic is often used. A total volume of 1.5–2 ml is recommended to avoid a high spinal block. An important concern, especially in the elderly, with the use of spinal morphine is that of delayed respiratory depression. Patients receiving spinal morphine need to be monitored closely for the first 24 h.

Intravenous lidocaine infusion in the perioperative period decreases intraoperative anesthetic requirements, lowers pain scores, reduces postoperative analgesic requirements, improves return of bowel function and decreases length of hospital stay [73]. The analgesic benefits are seen in open and MIS and may last beyond the infusion. The literature is unclear whether the infusion should be continued postoperatively. Systemic toxicity is a rare but a potentially life-threatening complication. Symptoms include blurred vision, dizziness, tinnitus, perioral anesthesia and tongue paresthesia. All patients on a lidocaine infusion must be on a continuous ECG monitor for the duration of the infusion [73].

Abdominal wall blocks include transversus abdominal plane (TAP), subcostal and rectus blocks. TAP blocks provide analgesia from T10 to L1, i.e., below the umbilicus while subcostal and rectus blocks prove useful adjuncts to provide a block for the upper abdomen. Recent systematic reviews have shown that TAP blocks have shown reduced opioid consumption, earlier return of bowel function and shorter LOS in abdominal and gynecologic surgery [82, 83]. A concern is the short duration (8–10 h) of TAP blocks. Various methods are used to increase the duration of TAP blocks, these include infusion catheters and more recently liposomal bupivacaine [84].

Paracetamol and non-steroidal anti-inflammatories (NSAID’s) are key drugs in the perioperative period as part of a multimodal regimen. Opioids should be avoided, and short-acting opioids should be used if needed. Paracetamol is relatively inexpensive, has minimal side effects and is available in both oral and intravenous formulations. NSAIDs are equally useful in postoperative analgesia. However, the concern of an increased risk of an anastomotic leak remains, but recent literature and meta-analysis remain inconclusive [85, 86].

Given the different skill set, training, experience and variable resources and postoperative monitoring facilities, the modalities of treatment used needs to be tailored to the individual institution. The ability to provide the most effective opioid sparing analgesia that is safe and allows for early mobilization and oral feeding, remains the ideal approach.

Summary:

TEA analgesia in open abdominal surgery is recommended.

**Table:** 

Evidence	high
Recommendation	strong

Spinal analgesia in laparoscopic surgery is recommended.

**Table:** 

Evidence	moderate
Recommendation	strong

Abdominal wall blocks are recommended.

**Table:** 

Evidence	moderate
Recommendation	strong

The combination of paracetamol and NSAIDS are recommended as baseline multimodal analgesics in the postoperative period unless specific contraindications exist.

**Table:** 

Evidence	moderate
Recommendation	strong

#### Fluid balance

The key aim of intravenous fluids (IVF) is to maintain intravascular volume to ensure adequate tissue and organ perfusion and to avoid electrolyte imbalances. There is a narrow range of optimal fluid therapy since both over and under hydration can cause complications. Optimal preoperative fluid intake and avoiding bowel preparation will in most cases ensure the patient arrives in hospital well hydrated. Intraoperatively most patients will require balanced crystalloids (e.g., Ringer’s lactate) [87, 88]. The use of 0.9% saline should be avoided due to the risk of salt and fluid overload [87].

Oliguria should not trigger fluid therapy as low urine output is a normal physiological response during surgery and anesthesia and could be due to multiple factors. Oliguria should not be managed in isolation but rather be investigated and the cause established prior to additional fluid therapy [87–89].

Postoperatively, IVF should be discontinued at latest during day1. Patients should be encouraged to drink when fully recovered and offered an oral diet within 4 h after abdominal/pelvic surgery [87]. If IVF’s need to be continued postoperatively a hypotonic crystalloid with 70–100 mmol/day of sodium and up to 1 mmol/kg/day of potassium should be used [87]. Any ongoing losses (diarrhea, vomiting) needs to be replaced with a balanced solution (e.g., Ringer’s lactate) as required whereas 0.9% saline solutions avoided [88–90].

Summary:

Near zero fluid balance is recommended.

**Table:** 

Evidence	high
Recommendation	strong

Balanced salt solutions for maintenance and replacement are recommended.

**Table:** 

Evidence	low
Recommendation	strong

#### Minimally invasive surgery (MIS)

The benefits of MIS are well documented, and its use continues to increase worldwide [91, 92]. MIS may well have a wider applicability as a diagnostic procedure in LMIC’s, given the lack of access to radiology services [93]. MIS is associated with a decrease in intraoperative blood loss, reduced analgesic requirements, early return of bowel function, decreased complications (including SSI and incisional hernias) and shorter length of stay [91]. MIS also assists in the implementation of some of the key components of the ERAS care pathway: opioid sparing analgesia, optimal fluid therapy and early mobilization.

Minimally invasive surgery is preferred for appropriate patients where the resources and expertise are available. MIS in most primary and secondary hospitals in LMIC’s is not readily available due to a combination of a lack of equipment and adequate training. If the equipment and or expertise do not exist an open procedure will be the safer approach. As healthcare resources improve the training of the surgical team in MIS, the acquisition of the necessary equipment will need to be prioritized.

Summary: MIS should be used when the appropriate skills, equipment and a trained team is available.

**Table:** 

Evidence	high
Recommendation	strong

#### Avoidance of postoperative nasogastric tubes and drains

A nasogastric tube has traditionally been used routinely in abdominal surgery with the aim of reducing postoperative vomiting, ileus and gastric distension. A meta-analysis of RCT’s, and a subsequent Cochrane review confirmed no benefit and a significantly higher risk of postoperative atelectasis, pneumonia, pharyngitis and a delayed return of bowel function [94, 95]. An orogastric tube is recommended if the stomach was inadvertently inflated with endotracheal intubation but should be removed before reversal of the anesthetic.

A drain in the pelvis or abdomen has been used to drain either blood or serous fluid that may accumulate postoperatively and possibly prevent or detect an intra-abdominal collection or anastomotic leak. Multiple studies, including a recent a meta-analysis of 11 RCT’s, concluded that the use of pelvic and abdominal drains did not decrease anastomotic leak rates, reoperation or mortality [96].

Summary:

Avoiding routine nasogastric tube placement is recommended.

**Table:** 

Evidence	strong
Recommendation	strong

Avoid the routine use of peritoneal and pelvic drains is recommended.

**Table:** 

Evidence	strong
Recommendation	strong

#### Early postoperative feeding

Patients have traditionally been kept fasting postoperatively until signs of return of bowel function. However, prolonged fasting is associated with increased risk of postoperative infectious complications and delayed recovery. A Cochrane review has shown that early oral feeding (fluids immediately after surgery and solids after 4 h), when combined with measures to reduce postoperative ileus, is associated with earlier return of bowel function, a shorter length of hospital stays and does not pose a higher risk of anastomotic leaks and other complications [97]. Even with early feeding, oral intake seldom reaches the required energy or protein requirements. Oral nutritional supplements have been shown to be useful in both the well and under nourished patient [98].

The metabolic stress induced by surgery results in a depletion of immune modulators. The supplementation of oral feeds with immune modulators (immune-nutrition) is associated with a reduction in infective complications and a reduced length of stay [99]. The ESPEN guidelines, based on multiple RCT’s and meta-analysis, recommend that malnourished patients undergoing cancer surgery should at least receive postoperative immune-nutrition with arginine, omega 3 fatty acids and ribonucleotides [100]. However, the quality of evidence remains low. The recommendation is graded as weak for primary and secondary hospitals in LMIC’s and is not recommended.

Summary: Early oral feeding is recommended.

**Table:** 

Evidence	moderate
Recommendation	strong

#### Early mobilization

Early mobilization is an essential component of perioperative care to improve patient recovery after surgery. The duration of mobilization (hours/day) is uncertain. The current recommendation is for the patient to sit out of bed for 30 min on day 0 and 6 h/day thereafter and to commence walking on day1. While there is evidence to show increased risk of complications (increased muscle loss, pulmonary and VTE) with prolonged bed rest [101, 102], there is limited evidence on the benefits of a dedicated interventions to improve early mobilization [103]. The duration of mobilization (hours/day) is uncertain. The current recommendation is 30 min on day 0 and 6 h/day thereafter. Patients and their families should be educated on these goals preoperatively.

Summary: Early mobilization is recommended.

**Table:** 

Evidence	moderate
Recommendation	strong

#### Urinary catheter

A urinary catheter is traditionally placed prior to major abdominal or pelvic surgery to avoid urinary retention, improve patient comfort and to measure urine output. However, there is very little evidence to support this practice. The placement and the duration of an indwelling catheter is associated with a higher risk of urinary tract infection (UTI). UTI is the fourth leading cause of all hospital-acquired infections and leads to increased hospital costs, length of stay, and risk of mortality [104, 105]. Other complications include, hematuria, catheter blockage, patient discomfort and delayed mobilization. Urinary retention is uncommon. In a recent large observational study, in an ERAS program, only 14% of patients developed retention [106].

Summary: Foleys catheter should be removed in the majority of cases within 24 h after surgery and individualized in patients with high risk of retention.

**Table:** 

Evidence	high
Recommendation	strong

#### Tailored postoperative monitoring, evaluation and escalation of care

Postoperative mortality is higher following surgery in LMIC’s than in HIC [4, 5]. Failure to rescue (FTR), defined as the death of a patient following one or more complications, is thought to be a key contributor [107].

Patients frequently suffer an index complication resulting in further complications which may ultimately end in the death of the patient. Not all complications can be prevented, but timely recognition and appropriate intervention may reduce mortality. Depending on the patient population, FTR rates vary from less 1% to over 40% despite similar complication rates [108]. This would suggest that many lives could be saved through the early identification, escalation of care and management of postoperative complications. Early warning scoring systems, such as the manual National Early Warning System (NEWS) and Modified Early Warning System (MEWS), and the electronic Cardiac Arrest Risk Triage (eCART), have been developed to risk stratify inpatients who are at risk for adverse events and have been shown to be of benefit in HIC [109, 110]. Patient and family initiated rapid response teams to escalate management has also shown to be of benefit [111]. These tools to reduce FTR are yet to be adequately tested in under resourced LMIC’s [112]. Combing these tools with the ERAS program with its focus on multimodal opioid sparing analgesia, early transition from IV to oral fluids and early mobilization may be a useful in identifying the at risk patient. Although intuitively appealing this will need to be tested before it can be widely implemented.

Postoperative monitoring, evaluation, escalation of care and FTR is a complex entity. The causes of FTR are multifactorial and any plan needs to adopt a multidisciplinary approach to identify the macro- and microsystem factors at play. Primary and secondary hospitals in LMIC’s will need to identify their patient and institution risks and develop a clear, concise, tailored, postoperative monitoring, evaluation and escalation pathway. Key parameters to measure and monitor include respiratory and heart rate, blood pressure, oxygen saturation, level of consciousness and surgical site. Use of some type of standardized early warning score in conjunction with an escalation protocol for deteriorating scores, will help identify at risk patients and promote rescue.

Summary: Institutional specific guidelines for postoperative monitoring, evaluation and escalation of care is recommended.

**Table:** 

Evidence	moderate
Recommendation	strong

#### Audit and evaluation

Audit against defined standards is a central component of quality improvement. Simple audit can be performed using paper checklists and tracking observed performance against standards. More sophisticated evaluation and monitoring tools facilitate quality improvement by providing a platform to audit compliance to best practice, benchmark patient outcomes and inform policy. Auditing compliance to the guidelines is a key component of the ERAS program to ensure improvement in quality of care. Improved compliance is associated with a decrease in length of stay and complications [7, 113]. There are many different platforms to collect and review data. The limitations of most of these platforms is the lack of uniformity in definitions, data fields that are captured and difficulty with data verification. The ERAS Society and ERAS USA have published the Reporting on ERAS Compliance, Outcomes and Elements Research (RECOvER) Checklist for reporting compliance and clinical pathways [114]. The ERAS Society has developed an ERAS Interactive Audit system that is used together with a dedicated implementation program to effect sustainable change, benchmarking and research. Auditing compliance to the guidelines and monitoring outcomes is an essential component of the ERAS program.

Summary: Regular audits of compliance to guidelines and reporting of outcomes is recommended.

**Table:** 

Evidence	high
Recommendation	strong

## Conclusion

These guidelines, a collaboration with established leaders in perioperative care from the ERAS Society and low- and middle-income countries, represent a collaborative effort to establish contextually relevant guidelines and to facilitate the delivery of high quality, equitable perioperative care.

The key objective of this work is to provide broad-based guidelines for elective abdominal and pelvic surgery in primary and secondary hospitals in LMIC’s. This is a significantly resource-constrained environment and surgical procedures and anesthesia is often carried out by generalists working across multiple specialties. Although we have been largely guided by the evidence from perioperative care for colorectal and gynecology, we do believe that these guidelines can be used for other elective abdominal procedures (e.g., small bowel resection, cholecystectomy, ventral wall hernia repair).

The guidelines care items, apart from FTR, are patient rather than system centric and will need to be implemented with a full understanding of the macro- and microsystem factors that impact on patient outcomes. Given the gravity of FTR in LMIC’s, we believe it is important to highlight the problem. However, its complexity together with the absence of robust data from LMIC’s specific recommendations cannot be made at this stage and individual institutions will need to develop a tailored evaluation and escalation of care plan.

These guidelines are seen as a starting point to address the urgent need to improve perioperative care and to effect data-driven, evidence-based care in LMIC’s. A limitation of this work that needs to be addressed in future guidelines would be to achieve a broader level of engagement of all stakeholders, a wider LMIC representation and to consider a single guideline that is adaptable to both HIC and LMIC’s.

The next step of this project is to pilot these recommendations using a structured implementation program and a standardized monitoring and evaluation tool. There are several studies including a Cochrane review that has demonstrated that as adherence (compliance) to ERAS guidelines improves (approximately 70% or greater), LOS and complications decrease and that there is a synergistic effect when the care items are implemented as a bundle [115–117]. Effective implementation and continuous audit of process measures are key to ensure improvement in quality of care and patient outcomes [118]. Once feasibility and efficacy are established, scale up can be planned.
